# Measurement of the Rates of Synthesis of Three Components of Ribosomes of *Mycobacterium fortuitum*: A Theoretical Approach to qRT-PCR Experimentation

**DOI:** 10.1371/journal.pone.0011575

**Published:** 2010-07-14

**Authors:** Maria Jesus Garcia, Maria Carmen Nuñez, Robert Ashley Cox

**Affiliations:** 1 Departamento de Medicina Preventiva, Facultad de Medicina, Universidad Autónoma, Madrid, Spain; 2 Division of Mycobacterial Research, National Institute for Medical Research, London, United Kingdom; Center for Genomic Regulation, Spain

## Abstract

**Background:**

Except for the ribosomal protein L12 (rplL), ribosomal proteins are present as one copy per ribosome; L12 (rplL) is unusual because it is present as four copies per ribosome. Thus, the strategies used by *Mycobacterium fortuitum* to regulate ribosomal protein synthesis were investigated, including evaluations of the rates of chain elongations of 16S rRNA, rplL and ribosomal protein S12 (rpsL).

**Methodology:**

RNA was isolated from cell cultures and cDNA was prepared. The numbers of cDNA copies of 16S rRNA, precursor-16S rRNA and transcripts of *rps*L and *rpl*L were quantified by qRT-PCR and then related to the rates of 16S rRNA, rpsL and rplL chain elongations by means of a mathematical framework for coupled transcription/translation.

**Principal Findings:**

The rates of synthesis of 16S rRNA, *rps*L and *rpl*L respectively were found to be approximately 50×10^3^ nucleotides h^−1^, 1.6×10^3^ amino acid residues h^−1^ and 3.4×10^3^ amino acid residues h^−1^. The number of transcripts of *rplL* was approximately twice that of *rpsL*. These data account for the presence of one copy of rpsL and four copies of rplL per ribosome, and reveal that the rate of *M. fortuitum* ribosome synthesis was closer to that of *M. tuberculosis* than to *E. coli*. Except for rplJ, the elongation rate obtained for rpsL was inferred to be appropriate for all other proteins present as one copy per ribosome.

**Significance:**

The results obtained provide the basis for a comprehensive view of the kinetics of ribosome synthesis, and of the ways that bacterial cells utilize genes encoding ribosomal proteins. The methodology also applies to proteins involved in transcription, energy generation and to bacterial proteins in general. The method proposed for measuring the fidelity of cDNA preparations is intrinsically much more sensitive than procedures that measure the integrity of 16S rRNA.

## Introduction

Mycobacteria are a group of bacilli that can be isolated from a wide range of environmental conditions [1]. Some are human pathogens, for example *Mycobacterium tuberculosis*. The mycobacteria are characterized by several remarkable features, such as their acid fast staining, their slow growth and their minimum content of (one or two) ribosomal operons per genome [1], [2]. The genus has been subdivided into two groups according to the time required to visualize colonies in solid media (more or less than seven days). The opportunistic pathogen, *Mycobacterium fortuitum* belongs to the group of rapidly growing mycobacteria (those mycobacteria that develop colonies in less than seven days). Members of this group usually have two copies of the *rrn* operon per genome [3].

Although the study of mycobacteria has intensified over the past twenty years there are few data for either their macromolecular compositions or for the rates at which their macromolecules are synthesized (for reviews see [4], [5]). However such information would help to further understand mycobacterial growth and the different growth rates shown by members of the genus.

A cell's capacity for protein synthesis is reflected in its RNA content [6]. For this reason the RNA fraction of a cell reflects the cell's metabolic activity. Techniques such as qRT-PCR can be used to study cell metabolism provided that the composition of the required cDNA preparation accurately reflects the composition of the RNA component of the population-average cell. The aim of our study was the analysis of the mycobacterial ribosome synthesis through the transcriptional study of three ribosomal components, namely, 16S rRNA and two ribosomal proteins rpsL (S12) and rplL (L7/L12).

The 16S rRNA moiety is the largest component of the small subunit of the ribosomes, it is encoded by the gene *rrs*, which is located near to the 5′-terminus of the *rrn* operon; *M. fortuitum* has two (*rrn*A and *rrn*B) *rrn* operons per genome [3]. The small subunit protein rpsL is involved in decoding the second and third positions of the codon at the A-site of the ribosome. Mutations in *rps*L confer resistance against streptomycin and can increase the accuracy of the decoding process, one copy of this protein is present per ribosome [7]. The large subunit protein rplL (L12) has a modified form (L7) which is acetylated at the N-terminus. A third large subunit protein rplJ (L10) together with two L7/L12 dimers form the stalk protuberance of the ribosome. L12 (including its acetylated form L7) is the only component of the ribosome that is present as more than one (specifically four) copies per ribosome. The functions of rplL (L7/L12) include involvement in translation factor binding, GTP-hydrolysis and translocation [7], [8]. Both *rps*L and *rpl*L are essential genes [9] and the encoded proteins are similar in size (125 and 131 amino acids respectively).

In this work, we investigated the rates of synthesis of three components of ribosomes during exponential growth of *M fortuitum*; namely, 16S rRNA and two ribosomal proteins rpsL (S12) and rplL (L7/L12). The numbers of transcripts of *rrs*, *rpl*L and *rps*L were quantified by qRT-PCR and then related to the rates of16S rRNA, rpsL and rplL chain elongations by using a theoretical framework for coupled transcription/translation. This framework was based on earlier studies [10], [11]. The results obtained provide the basis for a comprehensive view of the kinetics of mycobacterial ribosome synthesis.

## Results

**Figure 1:**
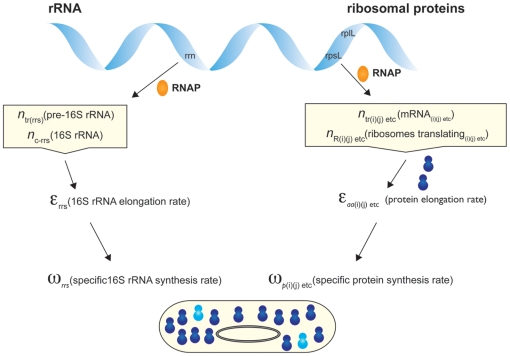
Scheme for the synthesis of rRNA and ribosomal proteins. The scheme shows the parameters of of the rRNA and protein components. The synthesis of rRNA is the rate limiting step in the synthesis of ribosomes. The synthesis of rRNA involves transcription of rRNA (*rrn*) operons as shown in Figure 2; the synthesis of ribosomal proteins is achieved by coupled transcription/translation (see Figure 3). The parameters used are defined in Table 1 and the required equations are listed in Table 2. The cartoon of a bacterium includes the genome and ribosomes that are represented in blue (newly synthesised ribosomes are shown in a lighter blue).

The theoretical framework followed for the synthesis of rRNA and ribosomal proteins (see the Theoretical Analysis section of Methods) is shown schematically in Figure 1. The required variables are given in Table 1. Equations (1) to (18) mentioned in Results are explained in the Theoretical Analysis section of Methods.

**Table 1:** Definitions of variables.

Symbol	Definition of variable (units in parenthesis)
*a*	Cell age [fraction of the generation time (time from birth/generation time); *a* = 0 for a newborn cell and *a* = 1 for a cell about to divide].
*l*_aa(i)_ ‡	Length (amino acid residues) of protein *p*_(i)_ encoded by ORF_(i)_.
*l*_p-rrs_	Length (nucleotides) of precursor-16S rRNA.
*n*_aa(av)_	Number of amino acid residues per population-average cell.
*n*_c-p(i)(av)_	Gross number of copies of protein *p*_(i)_ per population-average cell.
*n* ^#^_c-p(i)(av)_	Net number of copies of protein *p*_(i)_ per population-average cell.
n*_c-p(i)(av)_	Observed number of copies of protein *p*_(i)_ per population-average cell.
*n*_c-p(i)/R_	Gross number of copies of protein *p*_(i)_ per ribosome.
n*_c-rrs_	Number of copies of 16S rRNA per ng of the RNA substrate for cDNA synthesis.
*n*_c-rrs(av)_	Number of copies of 16S rRNA per population-average cell.
*n*_R(av)_	Number of ribosomes per population-average cell.
n*_R(i)_	Number of ribosomes translating n*_tr(i)_ transcripts of ORF_(i)_.
*n*_R(i)(av)_	Number of ribosomes per population-average cell actively synthesizing protein *p*_(i)_.
*n*_R(i)/tr(i)_	Number of ribosomes per transcript of ORF_(i)_ (see equation [18])
*n*_R(i)/ORF_	Number of ribosomes per ORF_(i)_ synthesizing protein *p*_(i)_.
*n*_RNAP(i)/ORF_	Number of RNAPs (RNA polymerase units) transcribing ORF_(i)_.
*n*_RNAP(rrs)(av)_	Number of RNAPs per population-average cell synthesizing precursor-16S rRNA.
*n*_RNAP(rrs)/ORF_	Number of RNAPs per gene synthesizing precursor-16S rRNA.
n*_tr(i)_	Number of transcripts of ORF_(i)_ per ng of RNA substrate for cDNA synthesis.
*n*_tr(i)/ORF_	Number of transcripts per ORF_(i)_.
*n*_tr(i)(av)_	Number of transcripts of ORF_(i)_ per population-average cell
n*_tr(rrs)_	Number of precursor-16S rRNA transcripts per ng of RNA substrate for cDNA synthesis.
*n*_tr(rrs)(av)_	Number of precursor-16S rRNA transcripts per population-average cell.
*α*	Footprint (base-pairs) of an initiating RNAP complex.
*β*	Footprint (nucleotides) of a ribosome.
*ε*_aa(av)_	Mean value of the peptide chain elongation rate (amino acids incorporated h^−1^) of the protein fraction of a population-average cell.
*ε*_aa(i)_	The peptide chain elongation rate (amino acids incorporated h^−1^) of protein *p*_(i)_.
*ε*_rrs_	The 16S rRNA chain elongation rate (nucleotides incorporated h^−1^).
*μ*	Specific growth rate (h^−1^).
*t*_D_	Duplication time.
*ω*_aa(av)_	The specific protein synthesis rate (amino acids incorporated h^−1^) of the protein fraction of a population-average cell.
*ω*_p(i)_	The specific protein synthesis rate (amino acids incorporated h^−1^) of protein *p*_(i)_.
*ω*_rrs_	The specific 16S rRNA synthesis rate (nucleotides incorporated h^−1^) per population-average cell.

‡, properties of proteins *p*_(i)_ and *p*_(j)_ respectively are indicated by the subscripts _(i)_ and _(j)_.

The main equations used for calculations are listed in Table 2.

**Table 2:** Equations used to evaluate 16S rRNA, rpsL and rplL chain elongation rates (see table 4 and tables S1 and S2).

Equation	Label in the text
*ε*_rrs_ = (n*_c-rrs_/n*_tr(rrs_) • *l*_p-rrs_ • *μ*	(6)
*ε*_aa(i)_ = (n*_c-rrs_/n*_tr(i)_) • (*n*_c-p(i)/R_/*n*_R(i)/tr(i)_) • *l*_aa(i)_ • *μ*	(13)
*n*_R(i)/tr(i)_ = (*α*+3*l*_aa(i)_)/2*β*	(18)
*n*_R(i)(av)_ = *l*_aa(i)_ *n*_R(av)_ *n*_c-p(i)/R_ (*μ*/*ε*_aa(av)_)	(10#)

Equation (6) was used to evaluate *ε*_rrs_; equations (13) and (18) were used to evaluate *ε*_aa(i)_; equations (18) and (10#) were used to evaluate n*_tr(i)_ for all ribosomal proteins of *E.coli* (*μ* = 0.42 h^−1^) cited in Tables S1 and S2.

Equation (10#) is readily derived by rearranging equation (10).

### The rate *ε*_rrs_ of 16S rRNA synthesis

**Figure 2:**
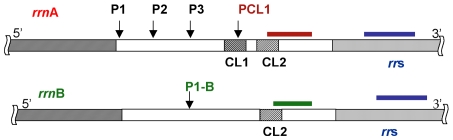
Schematic representation of the two *rrn* operons of *M. fortuitum*. Each operon comprises, in the order 5′ to 3′, the genes for 16S rRNA (*rrs*), 23S rRNA (*rrl*) and 23S rRNA (*rrf*). The 5′-end of the operon is presented. Horizontal bars indicate the regions of the transcripts analyzed using qRT-PCR: *rrs* (mature 16SrRNA) (blue); *rrn*A operon PCL1 (red) and *rrn*B operon P1-B (green). The detection of *rrn*A PCL1 includes all transcripts derived from all the four *rrn*A promoters (namely, P1 to P3 and PCL1). The rate *ε*_rrs_ nucleotides h^−1^ of 16S rRNA synthesis was calculated by means of equation (6). The analysis is based on two assumptions; first, that there is one precursor-16S rRNA per RNAP [34]; and secondly, that the synthesis of precursor-16S rRNA is completed before the synthesis of precursor-23S rRNA begins [33].

The organization of *rrn*A and *rrn*B are summarized in Figure 2. Operon *rrn*A has four promoters and *rrn*B has a single promoter [3]. The contribution of each *rrn* operon to the rRNA content can be measured by determining the amounts of the corresponding precursor rRNA (pre-*rrn*) transcripts [12]. Measurement of pre-*rrn*A can be determined by quantifying the number of transcripts due to the fourth *rrn*A promoter, namely PCL1 [2]. The results obtained for pre-16S rRNA synthesis are summarized in Table 3. The values of *ε*_rrs_ were calculated by means of equation (6). The average value and standard deviation were found to be 49,664±13,796 nucleotides h^−1^ (13.8±3.8 nucleotides s^−1^).

**Table 3:** Evaluation of 16S rRNA chain elongation rates (nucleotides h^−1^) during exponentially growth of *M. fortuitum*.

Experiment	cDNA	Optical density	*μ* (h^−1^)	*n* *_c-rrs_×10^−8^	*n* *_tr(A)_×10^−5^	*n* *_tr(B)_×10^−5^	*n* *_tr(rrs)_×10^−5^	*ε*_rrs_ #
1	(i)	0.62	0.026	6.29	4.79	0.49	5.28	56,450
	(ii)	0.62	0.026	4.00	7.17	0.66	7.83	24,200
2	(i)	0.69	0.026	6.18	6.34	0.66	7.00	41,823
	(ii)	0.69	0.026	5.59	3.92	0.39	4.31	61,441
3	(i)	0.95	0.026	5.66	5.75	0.54	6.29	42,627
	(ii)	0.95	0.026	7.53	5.66	0.57	6.23	57,257
4	(i)	2.45	0.038	3.59	2.08	0.16	2.24	(110,963)
	(ii)	2.45	0.038	3.00	4.95	0.36	5.31	39,116
5	(i)	3.61	0.038	4.46	4.22	0.24	4.46	69,236
	(ii)	3.61	0.038	8.60	10.35	0.51	10.86	54,828

(i) and (ii) denote independent cDNA preparations copied from different samples of the same RNA isolate. The variables are defined in Table 1.

*, denotes the number of transcripts measured by qRT-PCR per ng of RNA substrate used for cDNA synthesis.

16SrRNA chain elongation. n*_tr(A)_ and n*_tr(B)_ are respectively the number of transcripts of *rrn*A, transcripts of *rrn*B.

# , ε_rrs_ was evaluated by means of equation (6) where *l*_p-rrs_ = 1,822 nucleotide residues. The value enclosed in brackets is considered to be unreliable.

The average value found for *M. fortuitum* compares with a rate of 43,920 nucleotides h^−1^ (4–12 nucleotides s^−1^) reported for *M. tuberculosis* [13], the higher rate was inferred from the observation that the *rrn* operon (approx. 5,600 nucleotides) was synthesized in 7.6 minutes. The values *ε*_rrs_ cited for *E. coli* (*μ* = 0.42 h^−1^) range from 237,600 nucleotides h^−1^ (66 nucleotides s^−1^) obtained by Vogel & Jensen [14] to 306,000 nucleotides h^−1^ (85 nucleotides s^−1^) reported by Bremer & Dennis [15]. Thus, the value of *ε*_rrs_ that we have obtained is much closer to the value for *M. tuberculosis* than the value for *E. coli*. These data are consistent with the genus-specific properties of the mycobacterial transcriptional apparatus [16].

### Polypeptide chain elongation rates (*ε*_aa(i)_ and *ε*_aa(j)_) of rpsL and rplL

**Figure 3:**
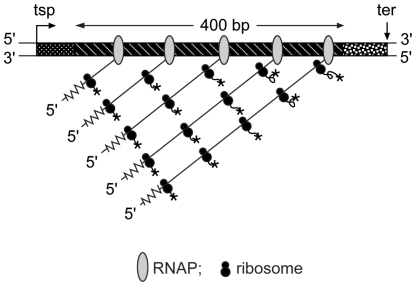
Scheme for coupled gene transcription/translation. The diagram represents a snapshot of an ORF of a population-average cell synthesizing protein. The size of the coding region (400 base-pairs) is close to that of *rps*L (375 base-pairs) and *rpl*L (393 base-pairs). The transcription start point (tsp), the 5′- and 3′-nontranslated regions and the terminus (ter) of transcription are indicated; RNAPs and ribosomes are not drawn to scale. RNAPs are spaced at one RNAP per 80 base-pairs and ribosomes at one ribosome per 80 nucleotides. The nascent polypeptide chains are shown as curly lines ending in an ‘*’. The instantaneous value of the specific synthesis rate of the encoded protein is the product of the number of ribosomes translating transcripts of the ORF and the polypeptide chain elongation rate (see equation (9)) The numbers of transcripts were measured by qRT-PCR and the numbers of associated proteins were deduced by means of the parameter *n*_R(i)/tr(i)_, the average number of ribosomes per transcript, which is by given by equation (18). The polypeptide chain elongation rate is given by equation (13). There is an upper limit to the numbers of transcripts and their associated ribosomes per ORF; for the example shown above the limit is five or so transcripts and fifteen or so ribosomes.

A scheme representing the coupled transcription/translation of an ORF of a population-average cell is shown in Figure 3. The data obtained for rpsL (subscript i) and rplL (subscript j) are summarized in Table 4. The values of *ε*_aa(i)_ and *ε*_aa(j)_ were evaluated by means of equation (13) using the terms *n*_R(i)/tr(i)_ = 2.84 and *n*_R(j)/tr(j)_ = 2.96 (see equation 18) which are appropriate for mycobacteria. The main result is that rplL is synthesized at a faster rate than rpsL, that is, *ε*_aa(i)_ = 1,640±1,050amino acid residues h^−1^ (0.46±0.30 amino acid residues s^−1^) and *ε*_aa(j)_ = 3,340±960 amino acid residues h^−1^ (0.93±0.26 amino acid residues s^−1^). The corresponding rate of synthesis of mRNA_(i)_ and mRNA_(j)_ chain elongations are inferred to be threefold faster; namely, 5,500 nucleotides h^−1^ and 10,065 nucleotides h^−1^ respectively which are 10% and 20% respectively of the value found for *ε*_rrs_ (Table 3). The two different ratios *ε*_aa(i)_ and *ε*_aa(j)_ reflect the fourfold difference in the number of copies per cell of rpsL and rplL. However, both proteins are similar in size; namely, 125 and 131 amino acid residues respectively. Consequently, the numbers of ribosomes per transcript (*n*_R(i)/tr(i)_ and *n*_R(j)/tr(j)_) are very similar (2.84 and 2.96 ribosomes/transcript respectively). Thus, the amplifications effects owing to transcription/translation coupling are very similar for both ORF_(i)_ and ORF_(j)_. It appears that the synthesis of rpsL and rplL in the ratio 1∶4 is achieved by a twofold increase in the number of transcripts of *rpl*L compared with *rps*L (Table 4) as well as twofold increase in *ε*_aa(j)_ compared with *ε*_aa(i)_ (Table 5).

**Table 4:** Evaluation of r-proteins polypeptide chain elongation rates (amino acids h^−1^) during exponential growth of *M. fortuitum*.

Experiment	cDNA	*n* *_tr(i)_×10^−5^	*n* *_R(i)_×10^−5^	*ε*_aa(i)_ ##	*n* *_tr(j)_×10^−5^	*n* *_R(j)_×10^−5^	*ε*_aa(j)_ ##
1	(i)	6.69	18.90	1,075	12.09	35.54	2,393
	(ii)	4.00	11.36	1,144	7.05	20.72	2,609
2	(i)	2.31	6.56	3,061	8.37	24.60	3,396
	(ii)	1.84	5.22	3,478	11.37	33.43	2,261
3	(i)	10.34	29.36	627	6.81	20.02	3,822
	(ii)	11.17	31.72	770	8.02	23.58	4,318
4	(i)	-	-	-	4.89	14.38	4,934
	(ii)	-	-	-	6.79	19.96	2,970
5	(i)	5.36	15.22	952	-	-	-
	(ii)	9.03	25.64	1,089	-	-	-

(i) and (ii) denote independent cDNA preparations copied from different samples of the same RNA isolate. The variables are defined in Table 1.

*, denotes the number of transcripts measured by qRT-PCR per ng of RNA substrate used for cDNA synthesis.

Polypeptide chain elongation. Corresponding values of optical density, μ (h^−1^) and n*_c-rrs_×10^−8^ are given in (A). The subscripts (i) and (j) refer respectively to rpsL and rplL. The parameters n*_R(i)_ and n*_R(j)_ were obtained from n*_tr(i)_ and n*_tr(j)_ respectively by multiplying by the factors *n*_R(i)/tr(i)_ = 2.84 and *n*_R(j)/tr(j)_ = 2.96 (equation 18).

## , *ε*_aa(i)_ and *ε*_aa(j)_ were evaluated by means of equation (13) where *l*_aa(i)_ = 125 and *l*_aa(j)_ = 131 amino acid residues, remembering that each ribosome has single copy of rpsL (*n*_c-p(i)/R_ = 1.0) and four copies of rplL (*n*_c-p(j)/R_ = 4.0).

**Table 5:** Comparison of the average numbers of transcripts of the genes studied per 1,000 copies of 16S rRNA and of the numbers of ribosomes per transcript.

Transcript	Number of Transcripts/1,000 copies of 16S rRNA	Number of ribosomes/transcript (*n*_R(i)/tr(i)_)
pre-*rrs*	1.19±0.42	Not applicable
*rps*L	0.75±0.50	2.84±0.5
*rpl*L	1.45±0.43	2.96±0.5

The average values were calculated from data presented in Tables 3 and 4. Equation (18) was used to evaluate *n*_R(i)/tr(i)_. See Table 1 for definitions.

Our analysis suggests that growth rate control of ribosome synthesis is governed not only by factors which include the rate(s) of polypeptide chain elongation of ribosomal proteins and parameters *n*_R(i)/tr(i)_, *n*_R(j)/tr(j)_ etc, which relate the requisite number of transcripts to the number of ribosomes needed to achieve the required protein synthesis rate, but also by the number of transcripts that the cognate ORFs can accommodate.

In principle, the ratio RNA∶protein can be deduced [17] from the ratio *ε*_aa(av)_ : *μ*. The ratio RNA∶protein may be deduced for *M. fortuitum* on the basis of the assumption that, by analogy with *E. coli*, *ε*_aa(i)_ provides an approximation for *ε*_aa(av)_. A value of 1 fg RNA: 3.42 fg protein was obtained by means of equation (19) which is based on the appropriate forms of equations (7) and (8). The fraction, *β*_R_, of ribosomes actively synthesizing protein is usually assigned a value of 0.8 [15].
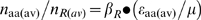
(19)The provisional value of *n*_R(av)_ = 4,000 (Table 5) is similar to the number of ribosomes reported for *M. bovis* BCG (*μ* = 0.03 h^−1^) [18] and corresponds to 9.7 fg RNA and hence to 40 fg protein per population-average cell. For comparison 58.7 fg protein per population-average cell was reported [19] for *Mycobacterium smegmatis* strain ATCC 607 and 100 fg protein per population-average was reported [15] for *E. coli* B/r (*μ* = 0.42 h^−1^).

### Comparison with *E. coli* and other bacteria

Population-average cells of *E. coli* B/r (*μ* = 0.42 h^−1^) have a complement of 6,800 ribosomes and their properties (Table 6) provide a frame of reference for the discussion of the results obtained for *M. fortuitum*. The rate of *E. coli* rRNA chain elongation was reported by Bremer & Dennis [15] to be 306,000 nucleotides h^−1^ (85 nucleotides s^−1^); and *ε*_aa(av)_ the average rate of polypeptide chain elongation was estimated [17] to be 43,200 amino acid residues h^−1^ (12 amino acid residues s^−1^). Examination of the *E. coli* model reveals that rplL is synthesised at a faster rate than rpsL (*ε*_aa(j)_>*ε*_aa(i)_). It was supposed (see Methods and Tables S1 and S2) that the average value for the protein fraction may be assigned to *ε*_aa(i)_; namely 43,200 amino acid residues h^−1^. As summarized in Table 6 the required rate of protein synthesis of rpsL is then achieved by 8.2 ribosomes translating 2.8 transcripts of *rpsL* per population-average cell. However, *ε*_aa(j)_ = 43,200 amino acid residues h^−1^ is not an appropriate rate for rplL synthesis which is present as four copies per ribosome. The peptide chain elongation rate of 43,200 amino acid residues h^−1^ requires 11.6 transcripts of *rplL* per population-average cell that are being translated by 32 ribosomes. The problem is that the population-average cell has 1.8 copies of *rplL* which can accommodate no more than 8.2 transcripts and 25.2 ribosomes. However, the required rate of rplL synthesis can be achieved if *ε*_aa(j)_ is equal to 2*ε*_aa(i)_ (86,400 residues h^−1^); then, the required 16 ribosomes translating 5.8 transcripts are readily accommodated by 1.8 copies of *rplL* per population-average cell. The number of transcripts per population-average cell of *rpsL* and *rplL* genes of *M. fortuitum* were found to be in step with their *E. coli* orthologues when the complement of ribosomes (*n*_R(av)_) was close to 4,000 (Table 6), as would be expected if bacterial cells utilize genes encoding ribosomal proteins in a characteristic way.

**Table 6:** Synthesis of ribosomes of *M. fortuitum* and comparison with *E. coli* B/r.

Ribosomal Component	Parameter	*M. fortuitum* *	*E. coli* †
	μ (h^−1^)	0.026	0.42
Ribosomes	*n*_R(av)_ (ribosomes)	4,000#	6,800
16S rRNA	*ε*_rrs_ (nucleotides h^−1^)	47,300±13,920	306,000
	*n*_RNAP(rrs)(av)_	4.00	17.2
	*n*_RNAP(rrs)(av)_/*n*_R(av)_	1.00×10^−3^	2.5×10^−3^
rpsL	*n*_c-p(i)/R_ (copies/ribosome)	1.0	1.0
	*ε*_mRNA(i)_ (nucleotides h^−1^)	4,575±3,280	129,600
	*ε*_aa(i)_ (amino acids h^−1^)	1,525±1,096	43,200
	*n*_R(i)(av)_ (ribosomes/cell)	8.52	8.2
	*n*_tr(i)(av)_ (transcripts/cell)	3.00	2.8
rplL	*n*_c-p(j)/R_ (copies/ribosome)	4.0	4.0
	*ε*_mRNA(j)_ (nucleotides h^−1^)	9,400±2,520	259,200
	*ε*_aa(j)_ (amino acids h^−1^)	3,150±840	86,400
	*n*_R(j)(av)_ (ribosomes/cell)	17.17	16.0
	*n*_tr(j)(av)_ (transcripts/cell)	5.80	5.8

Comparison of data calculated for *M. fortuitum* grown in Middlebrook 7H9 with *E. coli* B/r grown in succinate medium.

*, this study (see Tables 3 and 4).

# , estimated value (see text).

† , data for *E. coli* B/r [20]. The value for *n*_RNAP(rrs)_ was calculated using equations (1) and (2): *n*_R(i)(av)_ and *n*_R(j)(av)_ were calculated using equations (8) and (9); *n*_tr(i)(av)_ and *n*_tr(j)(av)_ were derived from *n*_R(i)(av)_ and *n*_R(j)(av)_ by means of *n*_R(i)/tr(i)_ and *n*_R(j)/tr(j)_. The value of *ε*_aa(av)_ was assigned to *ε*_aa(i)_ as reported for rpsA [23].

The calculations for both *S. coelicolor*A3(2) and *M. bovis* BCG are in accord with the data for *E. coli* B/r. The numbers of transcripts per cell were estimated to be 4.8 (0.5 per 1000 copies of 16S rRNA) and 9.6 (1 copy per 1000 copies of 16S rRNA) respectively for *rpsL* and *rpl*L for *S. coelicolor*A3(2) [20]. The numbers of transcripts per cell were estimated to be 1.3 (0.33 per 1000 copies of 16S rRNA) and 2.7(0.68 copies per 1000 copies of 16S rRNA) respectively for *rpsL* and *rpl*L for *M.bovis* BCG [18]. All three sets of data support the values found for *M. fortuitum*; the numbers of transcripts per cell were found to be 3.0 (0.75 copies per 1000 copies of 16S rRNA) and 5.80 (1.45 copies per 1000 copies of 16S rRNA) respectively for *rpsL* and *rpl*L.

### Appraisal of the methodology

The theoretical basis for evaluating the rate of synthesis of 16S rRNA is exact because it is based on the axiom that there is one RNAP per nascent transcript and no other considerations are involved.

The theoretical basis for evaluating polypeptide chain elongation rates is based not only on the axiom that there is one RNAP per transcript but also the parameter *n*_R(i)/tr(i)_ which is defined by the terms *α* and *β* in equation (18). In this study, it was estimated that the assigned values (*α* = 80 base pairs, *β* = 80 nucleotides) lead to values of *n*_R(i)/tr(i)_ that are better than plus or minus 20% (see Table 5).

Measurements of both RNA and protein chain elongation rates are critically dependent on the condition that the composition of the cDNA substrate accurately reflects the composition *in vivo* of the cognate RNA fraction of the population average cell (see equations (5) and (12)). The measured values of approximately one nascent transcript per 1000 copies of 16S rRNA (see Table 5) is in accord the data obtained for the reference species discussed in the previous section. These comparative data indicate that the conditions of equations (5) and (12) were met.

The gross numbers of copies of rpsL and rplL were each assumed to be the product of the number of copies per ribosome and the number of ribosomes per cell. With the exception of rpsJ, it was considered that ribosomal proteins were stable and that the pool of free proteins was negligible. In other words, the gross and net numbers of ribosomal proteins per cell were assumed to be equal. These assumptions were based on our knowledge of *E. coli* ribosomes; *E. coli* was the organism of choice for the study of bacterial ribosomes and protein synthesis. Many sequence similarities have been found between ribosomal RNA and ribosomal proteins of mycobacteria and their *E. coli* counterparts. Furthermore, mycobacterial ribosomes were found [21] to respond with equal facility to the factors IF1, IF2, IF3, EF-G, and EF-Tu irrespective of whether they were derived from *M. smegmatis* or *E. coli*. Thus elements of the translational machinery are highly conserved between both mycobacteria and *E. coli*. Formally, however, it is necessary to determine the numbers of copies of rpsL and rplL per population-average cell of *M. fortuitum*.

## Discussion

Little is known about the macromolecular compositions of mycobacteria or the rates at which their RNA and protein components are synthesized. In this work, qRT-PCR was used to measure the rates of synthesis of 16S rRNA, rpsL and rplL. The method is based on two assumptions: first, that the RNA of a bacterial cell reflects the cell's metabolic activity; and second, that the composition of each cDNA preparation accurately reflects the composition of the RNA component of cognate population-average cells.

Considering previous data, it is likely that the rates of polynucleotide chain elongation for 16S rRNA, 23S rRNA and 5S rRNA are all similar [22]. As expected, the rate of 16S rRNA elongation that we have obtained in *M. fortuitum* is much closer to the value for *M. tuberculosis* than the value for *E. coli* [13].

A feature of transcription/translation coupling (see equation (13)) is that *ε*_aa(i)_ the polypeptide chain elongation rate is largely independent of the size of the encoded protein. Thus, with the exception of rplJ, it is possible that the rates of polypeptide chain elongation may be similar for those proteins present as one copy per ribosome. Support for this proposal (see Tables S1 and S2) was obtained by applying equation (10#) (see Table 2) to data, including the polypeptide chain elongation rate for rpsA [23], relevant to ribosomal proteins of *E. coli*. The proposal may also be tested directly by using qRT-PCR to measure the polypeptide chain elongation rates of ribosomal proteins that differ in size; for example, rpsA (481 amino acid residues) and rpsL (125 amino acid residues). The application of equation (13) to data for *M. fortuitum* predicts that 0.82 transcripts of *rps*A per thousand ribosomes are needed to achieve a polypeptide chain elongation rate of 1,600 amino acids h^−1^, compared with 1.04 transcripts per thousand ribosomes needed for rpsL to achieve the same rate (see also Table S1).

Within the ribosome, rplL is known to form a stable pentameric complex comprising one copy of rplJ (L10) and four copies of rplL (L7/L12). This pentamer was shown to survive the procedures of both protein isolation and two-dimensional gel electrophoresis; the complex was first identified as L8. In contrast, rplJ was shown to be very rapidly degraded when not complexed with rplL [24].

The genes *rpl*J and *rpl*L form an operon in *E. coli*, *Streptomyces coelicolor* A3(2) and mycobacteria. The operon was found to have a single start site for transcription located upstream from rplJ in both *E. coli* [25] and *S. coelicolor* A3(2) [20]. Transcription was found to be autogenously controlled by the pentameric complex of one copy of rplJ with four copies of rplL binding to a site within the leader region of *rpl*J [26].

Examination of the genomic sequences available for mycobacteria revealed that the intergene regions between *rpl*J and *rpl*L ranged from 36 to 69 base pairs. The principal feature of this region is a strong Shine/Dalgarno sequence 5′AGGAAGGA 3′ located 8–10 nucleotides from the ATG start codon of *rpl*L (see Table S3). The above-mentioned sequence has the capacity to form 8 base pairs (including one G-U base pair) with the 3′-terminal sequence of 16S rRNA [27]. We infer that this interaction facilitates the efficient progress of ribosomes through the intergene region of the nascent transcript to begin synthesis of rplL. No other conserved sequence motif was identified in the intergene region plus the first five codons of *rpl* L; briefly, no evidence for a transcriptional enhancer was identified. Please note that the Shine/Dalgarno sequence upstream from *rpl*J was identified as 5′aggagg3′ which can form 6 (four g-c, and 2a-u) base pairs with the anti-Shine/Dalgarno sequence (see Table S3). Thus, the two ribosome binding sites found within the operon are similar in strength and are unlikely to have led to different rates for *rpl*J and *rpl*L transcription.

These data allow us to propose that *rpl*J and *rpl*L are transcribed at approximately equal rates leading to the synthesis of one copy of rplJ for each copy of rplL. Interaction between rplJ and rplL leads to the formation of the pentameric complex that is incorporated into ribosomes. The excess copies of rplJ synthesized are rapidly degraded, possibly by specific proteases. In brief, we propose that the ratio of four copies of rplL to one copy of rplJ is achieved by rapid and selective degradation of non-complexed rplJ. The pool of free copies of *E.coli* rplJ is small (3.5% [27]) compared with the much larger number (6800) of copies present within ribosomes. It was estimated (see Table S2) that only approximately 22 nascent polypeptide chains undergo synthesis and degradation at any instant. The proposal that the gross number and net number of copies of rplJ per cell are in the ratio of 4∶1 can be tested in the following way. The gross number of copies can be obtained by measuring the polypeptide chain elongation rate and the net number of copies can be measured empirically. If our proposal is correct the polypeptide chain elongation rates of rplJ and rplL should be equal. In contrast, the measured numbers of copies of these proteins should be in the ratio of one copy of rplJ to four copies of rplL.

Accurate procedures for RNA isolation and cDNA synthesis are crucial for the study of cell properties by qRT-PCR. Formally, it is necessary to show that the composition of cDNA copied from RNA *in vitro* accurately reflects the composition of the cognate RNA *in vivo*. We propose that the measurements of *ε*_rrs_, *ε*_aa(i)_, and *ε*_aa(j)_ provide a stringent test for the integrity of cDNA preparations because any reduction in the number of transcripts of *rrs*, *rpsL*, *rplL* through degradation etc would lead to erroneously high values of the chain elongation rates. Such a test is likely to be 500–1000 times more sensitive (Table 6) than an inspection of the profile of rRNA after electrophoresis in denaturing gels because *in vivo*, during exponential growth, the number of transcripts of the genes studied is approximately one thousandth of the number of copies of 16S rRNA (Table 5).

The results we have obtained for the rates of synthesis of 16S rRNA, rpsL and rplL provide the basis for a more comprehensive view of the rates of synthesis required for components of bacterial ribosomes (see Tables S1 and S2).

### Concluding remarks

Our study supports the notion that the metabolic activity of a bacterial cell is encapsulated in the RNA fraction of a cell culture represented by the population average cell. This information can be recovered provided that the required cDNA preparations accurately reflect the compositions of the cognate RNA fractions *in vivo*.

We have used the methods described above to recover values of the rates of extension of 16S rRNA and two ribosomal proteins by studying normal cell metabolism. Earlier methods have relied on procedures that may perturb normal cell metabolism; for example, drugs such as rifampicin [13], [28], radioactive tracers [13] and the introduction of engineered plasmids carrying reporter genes [14], [23] have all been used.

There have been few studies of the rates of synthesis of specified proteins. For example; although the stoichiometry of ribosomal proteins has been known for more than thirty years, until now there has been no explanation of the way in which the bacterial cell regulates protein synthesis in order to provide the ribosome with four copies of rplL and one copy of all other component proteins. Our results support the view that rplL synthesis requires higher transcription and translation rates than other ribosomal proteins.

In principle, the theoretical analysis we have described has the potential for measuring the gross number of copies of a specified protein per cell; a parameter that is not readily accessible. For example, for any protein *p*_(i)_, an independent measurement of *ε*_aa(i)_ would allow the gross number *n*_c-p(i)/R_ of copies of protein *p*_(i)_, per ribosome to be evaluated (as can be inferred from equation 13). The number of copies of *p*_(i)_ per cell may then be calculated once the number of ribosomes per cell becomes available. Comparisons of gross and empirical values of the numbers of copies of a protein could be informative about the extent of its degradation or export.

There is a need for accurate and reliable measurements of the macromolecular compositions (DNA∶RNA∶protein) of cell cultures in order to provide the basis for quantitative studies of cell metabolism. As we have shown (see Tables S1 and S2) such information may provide the means for testing new proposals for metabolic processes.

Finally, the availability of complete genomic sequences allows us to anticipate the emergence of views of bacterial growth and development that are both quantitative and dynamic; this report describes an early step in this direction.

## Methods

### Theoretical analyses

The variables considered are defined in Table 1.

#### Population-average cells

Schaechter *et al.* [6] proposed that for exponentially growing cells an average number (*n*_x(av)_) of constituent, *x*, per cell may be defined as the number of *x* per ml of culture divided by the number of cells per ml of culture. The population-average cell defined in this way reflects the properties of the entire cell population, which includes cells of all ages, *a*, ranging from newborn cells (*a* = 0) to cells about to divide (*a* = 1). Quantities measured by qRT-PCR in the study of an RNA fraction isolated from a cell culture may now be defined in terms of parameters of population-average cells which are denoted by the subscript (av) [10], [11].

#### Ribosomal components are located mainly in ribosomes

Bacterial ribosome synthesis is governed by a co-ordinated production of individual ribosomal components. Synthesis of rRNA is a highly regulated response to the nutrients available to the cell (for review see [22]). The synthesis of ribosomal proteins (r-proteins) is tightly linked to rRNA synthesis by feedback inhibition, termed autogenous control (for reviews see [7], [29], [30], [31]). Autogenous control of r-protein synthesis suggests that within the cell r-proteins are located mainly in the ribosomes. This expectation was shown to be correct [32]; apart from rpsA and rpsB, pools of free r-proteins were found to be less than 7% of the total number of copies. Furthermore, the pool size of rpsL was found to be within background levels whereas the pool size of rplL was 0.5% and of rplJ was 3.5%. Pools sizes can be taken into account by the inclusion of an appropriate factor (for example, 1.035 in the case of rplJ) in equation (8) below.

#### Stability/degradation of ribosomal proteins

Ribosomes are stable enzymes catalyzing sequential peptide bond formation with lifetimes that exceed the lifespan of individual cells. The pool sizes of free component proteins are small (see the preceding Section), r-protein synthesis is subject to autogenous control (see references 7,29–31), newly synthesized component proteins are free to interact with nascent precursor 16S rRNA and 23S rRNA to form nascent 30S and 50S ribosomal subunits; in contrast, ribosomes are abundant within the cell. For these reasons we concluded that degradation of r-proteins is not a significant factor in the measurement of their polypeptide chain elongation rates.

The mathematical analysis below is based on the assumption that pools of ribosomal proteins are small so that the numbers of copies per cell of the three components studied (16S rRNA, rpsL and rplL) are each equal to the product of the number of ribosomes per cell and the number of copies of the component per ribosome.

#### The rate of 16S rRNA synthesis

*M. fortuitum* has two rRNA operons (*rrn*A and *rrn*B). The promoter regions and the 5′-ends of the 16S rRNA (*rrs*) genes of the two operons are shown in (Figure 2). Each operon has the classical structure of leader region, 16S rRNA coding region, Internal Transcribed Spacer region 1 (ITS1), 23S rRNA coding region, Internal Transcribed Spacer region 2 (ITS2), 5S rRNA coding region and trailer region [2], [3]. The above-mentioned studies show that the leader regions contained a highly conserved sequence motif Conserved Leader 2 (CL2) which is implicated in both antitermination of transcription and the formation of precursor-16S rRNA (pre-16S rRNA). A complementary sequence cCL2 is located downstream from the 3′-end of the 16S rRNA coding region. The interaction of CL2 and cCL2 sequence motifs leads to the formation of a double-helical stem structure that contains an RNAaseIII processing site. Cleavage at this site generates pre-16S rRNA [3]. The study of the transcription of *E. coli rrn* operons by electron microscopy [33] revealed a double ‘Christmas tree’ effect that showed that cleavage of the pre-16S rRNA gene took place before the transcription of the 23S rRNA gene began. This information, together with the axiom [34] that each “RNA polymerase (RNAP) complex synthesizing RNA generates a single RNA chain”, provides the basis for the measurement of *ε*_rrs_ nucleotides h^−1^, the 16S rRNA chain elongation rate, by qRT-PCR.

During exponential growth *ω*_rrs_ the specific synthesis rate of pre-16S rRNA is given by equation (1) where *n*_R(av)_ is the number of ribosomes per population-average cell, *l*_p-rrs_ is the length (nucleotides) of pre-16S rRNA and *μ* is the specific growth rate.
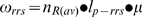
(1)However, *ω*_rrs_ is also the product of *n*_RNAP(rrs)_ the number of RNAPs synthesizing pre-16S rRNA and *ε*_rrs_ the rate of 16S rRNA chain elongation (see equation 2)
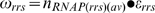
(2)Equating the right hand sides of equations (1) and (2) and making *ε*_rrs_ the subject leads to equation (3).
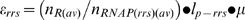
(3)Equation (4) applies because a ribosome has a single copy of 16S rRNA and one RNAP has a single transcript.
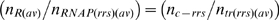
(4)Both n*_c-rrs_ and n*_tr(rrs)_, respectively the number of copies of 16S rRNA and the number of transcripts of precursor-16S rRNA per ng RNA substrate for cDNA synthesis, can be measured by qRT-PCR.
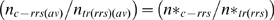
(5)Equation (5) is a formal statement of the requirement that the composition of the cDNA used in qRT-PCR experiments must accurately reflect the composition of the RNA component of the population-average cell. Provided that equation (5) applies *ε*_rrs_ may be evaluated by means of equation (6).
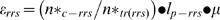
(6)


#### Transcription/translation of an individual ORF (ORF_(i)_)

Coupling between the processes of bacterial transcription and translation has long been accepted [35], [36]. A snapshot of an ORF undergoing transcription/translation was obtained by electron microscopy of lysates of fragile *E. coli* cells [33]. RNA polymerase complexes (RNAPs) were shown at intervals along the gene, with nascent mRNA transcripts increasing in size according to the position of the RNAP; and a proportionate number of ribosomes attached to each nascent mRNA. Understanding the snapshot provided by electron microscopy in quantitative terms is key to analysing the results obtained by qRT-PCR. It is not known how the bacterial cell achieves transcription/translation coupling although the transcription elongation factors NusA and NusG may play a role (for review see [37]). However, the rate of transcription must limit the rate of translation. Specifically, the rate of codon synthesis (*ε*_mRNA(i)_/3; where *ε*_mRNA(i)_ is the rate mRNA_(i)_ of chain elongation) must be less than the rate limiting step in peptide bond formation [38], [39]. When ribosomes translate a previously synthesized mRNA the rate limiting step is the interaction of a ternary complex of aminoacyl-tRNA, elongation factor EF-Tu and GTP with the A-site of the ribosome [40].

#### The rate of chain elongation of a ribosomal protein

The transcription/translation of a particular ORF (ORF_(i)_) is represented schematically by the fibril diagram shown in Figure 3. The ORF is shown to be fully loaded with RNAPs and transcripts loaded with ribosomes. A population-average cell is representative of a very large number of cells of different ages [10]. For a single copy gene the number of fibrils per population-average cell will depend on age of the cell when the ORF_(i)_ is replicated; this number cannot exceed two and is more likely to approximate to 1.5. Thus, there is an upper limit per cell for both *n*_tr(i)(av)_ the number of transcripts of ORF_(i)_ and *n*_R(i)(av)_ the number of ribosomes synthesizing the encoded protein *p*_(i)_.

The specific synthesis rate, *ω*_p(i)_, of protein *p*_(i)_ is given by equation (7) where *n*_c-p(i)/c_ is the number of copies of *p*_(i)_ per cell and *l*_aa(i)_ amino acids is the length of *p*_(i)_.

The number of copies of protein *p_(i)_* per population-average cell is affected by the extent to which the protein is either degraded or exported. Thus, it is necessary to distinguish between the gross value *n*_c-p(i)(av)_, the net value *n^#^*_c-p(i)(av)_ and the empirical value *n* ^*^_c-p(i)(av)_ of the number of copies of protein *p*_(i)_ per cell. When there is neither degradation nor export of the protein the gross, net and empirical values are equivalent. When the protein is degraded or is exported the gross value is greater than the net value which is equal to the empirical value of the number of copies per cell.
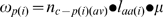
(7)The number of copies of *p*_(i)_ per cell is the product of *n*_R(av)_ and *n*_c-p(i)/R_ the gross number of copies of protein *p*_(i)_ per ribosome. Substitution for *n*_c-p(i)(av)_ in equation (7) leads to equation (8).
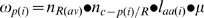
(8)The specific protein synthesis rate of *p*_(i)_ is also equal to the product of *n*_R(i)(av)_ the number of ribosomes per cell synthesizing *p*_(i)_ and *ε*_aa(i)_ the rate at which the peptide chain is elongated (see equation 9).
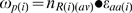
(9)Equating the right hand sides of equations (8) and (9) and making *ε*_aa(i)_ the subject leads to equation (10).
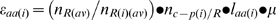
(10)
*ε*_aa(i)_ may be calculated by qRT-PCR in the following way: The number *n*_R(av)_ of ribosomes per cell is equal to *n*_c-rrs_ the number of copies of 16S rRNA per cell. The number *n*_R(i)(av)_ of ribosomes synthesising *p*_(i)_ per cell can be evaluated through the parameter *n*_R(i)/tr(i)_ which relates *n*_R(i)(av)_ to *n*_tr(i)(av)_ the number of transcripts of ORF_(i)_ per population-average cell (see equation 11).
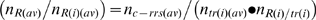
(11)The experimentally accessible parameters are n*_c-rrs_ and n*_tr(i)_ which are respectively the numbers of copies of 16S rRNA and numbers of transcripts of ORF_(i)_ per ng of RNA used as the substrate for cDNA synthesis. Thus, *ε*_aa(i)_ may be evaluated provided that the composition of the cDNA substrate for qRT-PCR accurately reflects the composition of the RNA component of the population-average cell, as stated in equation (12).
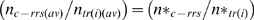
(12)Hence equation (13), which links the number of transcripts of a particular ORF that are needed for protein synthesis with the gross number of copies of the encoded protein per ribosome, allows the polypeptide chain elongation rate to be evaluated.
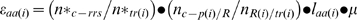
(13)


#### Evaluation of the number of ribosomes per transcript

We define the conversion factor *n*_R(i)/tr(i)_ as *n*_R(i)/ORF_ the number of ribosomes translating transcripts of ORF_(i)_ divided by *n*_tr(i)/ORF_ the number of transcripts per ORF_(i)_. There is one transcript per RNAP so that *n*_R(i)/tr(i)_ the number of ribosomes per transcript is equal to the number of ribosomes per RNAP (see equation 14).
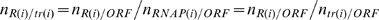
(14)ORF_(i)_ will be fully loaded with RNAPs when, on average, there is one RNAP per ***α*** base-pairs of ORF_(i)_ (see equation 15).
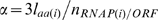
(15)The maximum number of RNAPs per ORF is determined by the footprint of the initiating RNAP at the promoter. Krummel & Chamberlin [41] found that the initiating complex of an RNAP at the promoter had a footprint of about 80 base-pairs; that is, ***α*** approximately 80 base-pairs.

Furthermore, the lengths of transcripts associated with adjacent RNAPs will differ by ***α*** base-pairs. Suppose that there is one ribosome per *β* nucleotides of mRNA: then the number of ribosomes per transcript will increase according to an arithmetic progression. The sum of the progression yields *n*_R(i)/ORF_ which is given by equation (16).
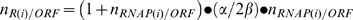
(16)Rearrangement of equation (16) leads to equation (17).
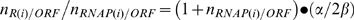
(17)Thus, by definition (see equation 14) *n*_R(i)/tr(i)_ is given by the right hand side of equation (17). Substitution for *n*_RNAP(i)/ORF_ (see equation 15) on the right hand side of equation (17), followed by simplification leads to equation (18).
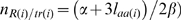
(18)The diameter of a bacterial ribosome [28] is 25 nm (250 Å). Neglecting secondary structure this dimension correspond the length of 74 nucleotides. It is inferred that, to a first approximation, *β* = 80 nucleotides [10]. Hence, *n*_R(i)/tr(i)_ can be evaluated by means of equation (18). Errors in the values of *α* and *β* are likely to be small so that values of *n*_R(i)/tr(i)_ are expected to be better than plus or minus 20%.

#### *Escherichia coli* B/r, *Streptomyces coelicolor* A3(2) and *Mycobacterium bovis* BCG as model systems

Data for the macromolecular compositions of *E. coli* B/r [15] and our knowledge of the *E. coli* genome provide sufficient information for the evaluation of parameters such as *n*_RNAP(rrs)_ (hence *n*_tr(rrs)(av)_) and *n*_R(i)(av)_ (hence *n*_tr(i)(av)_) to be achieved by means of the equations presented in Table 2. The theoretical values obtained provide a frame of reference for testing the validity of the results obtained by qRT-PCR for *M. fortuitum*.

Data for *E. coli* B/r (*μ* = 0.42 h^−1^) grown in succinate medium [15] were chosen because the genome is then replicated once only during the cell division cycle [15]. The relevant data are: *μ* = 0.42h^−1^; *n*_R(av)_ = 6800 ribosomes; *n*_aa(av)_ = 5.6×10^8^ amino acid residues; *ε*_aa(av)_ = 43,200 amino acid residues h^−1^; the lengths of rpsL, and rplL respectively are 124 and 121 amino acid residues.

For *E. coli* (*μ* = 0.42 h^−1^), *ε*_aa(av)_ the average rate of polypeptide chain elongation was estimated to be 43,200 amino acid residues h^−1^ (12 amino acid residues s^−1^) [17]. Several proteins involved in protein synthesis were found to have peptide chain elongation rates similar to the above-mentioned average value; the proteins studied were infB [14], tuf, rpsA (the largest r-protein) and fus [23]. These data suggest that at least to a first approximation of *ε*_aa(av)_ the average rate for the protein fraction may be assigned to all r-proteins present as a single copy per ribosome; as shown in Supplementary Data (see Tables S1 (proteins of the 30S ribosomal subunit) and S2 (proteins of the 50S ribosomal subunit)).

Data for *S. coelicolor* A3(2) [10], [20] and *M. bovis* BCG [18] were also used to provide reference data for the synthesis of rpsL and rplL of *M. fortuitum*. The data [10], [20] for *S. coelicolor* A3(2) are: *μ* = 0.024h^−1^; *n*_R(av)_ = 10500 ribosomes; *n*_aa(av)_ = 7.8×10^8^ amino acid residues; *ε*_aa(av)_ = 2,200 amino acid residues h^−1^; the lengths of rpsL and rplL respectively 123 and 124 amino acid residues. The data [18] for *M. bovis* BCG are: *μ* = 0.03h^−1^; *n*_R(av)_ = 3900 ribosomes; *n*_aa(av)_ = 4.03×10^8^ amino acid residues; *ε*_aa(av)_ = 3,900 amino acid residues h^−1^; the lengths of rpsL and rplL respectively 125 and 131 amino acid residues. In each case equation (13) was applied on the basis of the assumption that *ε*_aa(av)_ = *ε*_aa(i)_ and 2*ε*_aa(av)_ = *ε*_aa(j)_.

### Experimental design

#### Bacterial strain and RNA isolation

*M. fortuitum* ATCC 6841^T^ was grown at 37°C until exponential phase in medium Middlebrook 7H9 supplemented with Tween80 and ADC (Albumin, Dextrose and Catalase. Difco).

Mycobacterial cultures were collected, and total RNA isolated as described previously [42]. Standard precautions were undertaken to avoid contamination with RNases during the purification of total RNA [43]. Precautions taken in the manipulation of RNA included separate equipment and materials to be used only for experiments with RNA as well as the use of Diethylpyrocarbonate (Sigma) and RNasin (Promega). The integrity of the isolated RNA was checked by gel electrophoresis and RNA was quantified by spectrophotometry [43].

#### Analysis of mRNA by qRT-PCR

One hundred ng of the mycobacterial RNA isolated was reverse transcribed by using 30 U AMV reverse transcriptase and random primer hexamers (Promega). After DNase treatment the absence of DNA was confirmed by performing conventional PCR using the primers FoPCL1 and cKK4 [44].

Two independent cDNA preparations were obtained from individual samples of the same RNA isolate. For each cDNA preparation at least two independent qRT-PCR experiments were carried out usually in triplicate. Each qRT-PCR measurement cited in the Tables is the average of four or more determinations.

Real-time PCR was carried out using a capillary PCR instrument (Light Cycler; Roche). The oligonucleotides used in qRT-PCR are indicated in the Table 7. The amplification of the target sequence was detected using SYBR green. Conditions were used as follows: LightCycler Fast Start DNA master SYBR Green I reagent (1µl) was supplemented with 3.5 mM (final concentration) MgCl_2_ and 0.5 mM of each primer in 7µl of volume. Sample cDNA (3 µl) was added to the mix. The PCR cycling programme was as follows: denaturizing, 1 cycle of 95°C for 10 min with a transition rate of 20°C/s; amplification, 45 cycles at 95°C for 0 s, the corresponding annealing temperature for each product for 5 s and an extension at 72°C for 10 s with a single fluorescence acquisition, in all the cases the transition rate was 20°C/s. Specificity of the reaction was checked by analysis of the melting curve of the final amplified product. All PCR experiments were stopped before fluorescence was detected in the control negative capillaries.

**Table 7:** Primers and amplification conditions used in this study for qRT-PCR.

Primer	Target sequence	Sequence	Annealing Temp	Size amplicon
16S-F	rrs	5′…ATGACGGCCTTCGGGTTGTAA…3′	60°C	97 bp
16S-R		5′…CGGCTGCTGGCACGTAGTTG…3′		
L7-F	rplL	5′…TGGACGCGTTCAAGGAAATG…3′	62°C	74 bp
L7-R		5′…GACCTCGAAGGTCTCCTCGAA…3′		
PRS12-	rpsL	5′…CCACAACCTTCAGGAGCACT…3′	56°C	90 bp
PRS12-R		5′…GAACCCGCGATGATCTTGT…3′		
FoB10	P1-rrnB	5′…TTTTAGCCGCGGGATTTCT …3′	54°C	101 bp
FoB11		5′…AAGAGCGTGGCCAAAAAACA …3′		
PCL1Fort-F	PCL1-rrnA	5′…CAAAGCAGAAAAGCCTGTTG …3′	59°C	103 bp
PCL1Fort-R		5′…CAACAACCACACCCTAAACG…3′		

The qRT-PCR data were plotted as the fluorescence signal versus the cycle number. An arbitrary threshold was set at the midpoint of the log of fluorescent level versus cycle number plot. The Ct value is defined as the cycle number at which the fluorescent level crosses this threshold.

#### Macromolecular properties

Nucleotide sequence data for *rrn* operons were obtained as follows: gene bank entry accession number X99775 (*rrn*A) and see Menendez *et al.* [3] for *rrn*B. The lengths of rpsL and rplL were assigned values of 125 and 131 amino acid residues respectively by comparison to *M. tuberculosis* because these values are highly conserved within *Mycobacterium*.

## Supporting Information

Table S1 Synthesis of proteins of the 30S subunit of *E.coli* ribosomes calculated on the basis of transcription/translation coupling (see equations presented in Table 2). The abbreviations are defined in Table 1. lt ntr(i)(av) defines the limit (lt) to the numbers of transcripts per population average cell. The last column on the right is the fraction of the limiting (lt) number of transcripts needed to synthesize the required number (6800) of copies of the protein specified. The limiting number of transcripts is defined as the product of the number of copies of the specified ORF per cell and the maximum number (3 laa(i)/80) of transcripts per ORF. The number of copies of a particular ORF per cell was obtained by means of equation (9) of reference 15. The assumption that εaa(i) = εaa(av) = 43200 amino acid residues h−1 was based on the value reported [23] for rpsA when *E.coli* was grown at 370C in acetate medium (μ = 0.48 h−1.).
(0.07 MB DOC)

Table S2 Synthesis of proteins of the 50S subunit of *E.coli* ribosomes calculated on the basis of transcription/translation coupling (see equations presented in Table 2). The abbreviations are defined in Table 1. lt ntr(i)(av) defines the limit (lt) to the numbers of transcripts per population average cell. The last column on the right is the fraction of the limiting (lt) number of transcripts needed to synthesize the required number (6800) of copies of the protein specified. The limiting number of transcripts was estimated from the product of the number of copies of the specified ORF per cell and the maximum number (3 laa(i)/80) of transcripts per ORF. The number of copies of a particular ORF per cell was obtained by obtained by means of equation (9) of reference 15. Data for rplJ and rplL are explained in the main text.
(0.09 MB DOC)

Table S3 The Shine/Dalgarno motifs of *rpl*J (A) and the intergene regions (B) separating *rpl*J and *rpl*L of representative species of *Mycobacterium*. Sequences upstream from *rpl*J and intergene sequences are shown by lower case letters. The Shine/Dalgarno motifs and their binding sites are shown in bold italics. The light shading indicates secondary Shine/Dalgarno motifs.
(0.06 MB DOC)
