# Supplementary material for: Measurement of the Rates of Synthesis of Three Components of Ribosomes of Mycobacterium fortuitum: A Theoretical Approach to qRT-PCR Experimentation
Source: PLoS One. 2010 Jul 14;5(7):e11575. doi: 10.1371/journal.pone.0011575 (PMC2904383; doi:10.1371/journal.pone.0011575)
Supplement: Table S1 — Synthesis of proteins of the 30S subunit of E.coli ribosomes calculated on the basis of transcription/translation coupling (see equations presented in Table 2). The abbreviations are defined in Table 1. lt ntr(i)(av) defines the limit (lt) to the numbers of transcripts per population average cell. The last column on the right is the fraction of the limiting (lt) number of transcripts needed to synthesize the required number (6800) of copies of the protein specified. The limiting number of transcripts is defined as the product of the number of copies of the specified ORF per cell and the maximum number (3 laa(i)/80) of transcripts per ORF. The number of copies of a particular ORF per cell was obtained by means of equation (9) of reference 15. The assumption that εaa(i) = εaa(av) = 43200 amino acid residues h−1 was based on the value reported [23] for rpsA when E.coli was grown at 370C in acetate medium (μ = 0.48 h−1.). (0.07 MB DOC) [file pone.0011575.s001.doc]

**Table S1. Synthesis of proteins of the 30S subunit of *E.coli* ribosomes calculated on the basis of transcription/translation coupling (see equations presented in Table 2)**

| r-protein | *l*_aa(i)_ | *n*_R(i)/tr(i)_ | *ε*_aa(av)_ | *n*_R(i)(av)_ | *n*_tr(i)(av)_ | *lt n*_tr(i)(av)_ | *n*_tr(i)(av)_ /*lt* *n*_tr(i)(av)_ |
| --- | --- | --- | --- | --- | --- | --- | --- |
| rpsA (S1) | 557 | 10,9 | 43200 | 36,82 | 3,38 | 33,4 | 0,1 |
| rpsB (S2) | 241 | 5 | 43200 | 15,86 | 3,17 | 15,2 | 0,21 |
| rpsC (S3) | 233 | 4,85 | 43200 | 15,33 | 3,16 | 13,5 | 0,23 |
| rpsD (S4) | 206 | 4,3 | 43200 | 13,42 | 3,12 | 11,9 | 0,26 |
| rpsE (S5) | 167 | 2,68 | 43200 | 7,67 | 2,86 | 9,6 | 0,3 |
| rpsF (S6) | 131 | 3,03 | 43200 | 8,91 | 2,97 | 8,3 | 0,36 |
| rpsG (S7) | 179 | 3,37 | 43200 | 10,11 | 3 | 10,4 | 0,29 |
| rpsH (S8) | 130 | 2,92 | 43200 | 8,53 | 2,92 | 7,5 | 0,39 |
| rpsI (S9) | 130 | 2,92 | 43200 | 8,52 | 2,92 | 7,5 | 0,39 |
| rpsJ (S10) | 103 | 2,3 | 43200 | 6,8 | 2,96 | 5,9 | 0,5 |
| rpsK (S11) | 129 | 2,9 | 43200 | 8,13 | 2,8 | 7,4 | 0,38 |
| rpsL (S12) | 124 | 2,81 | 43200 | 8,13 | 2,89 | 7,2 | 0,4 |
| rpsM (S13) | 118 | 2,69 | 43200 | 7,73 | 2,87 | 6,8 | 0,42 |
| rpsN (S14) | 101 | 2,34 | 43200 | 6,48 | 2,77 | 5,8 | 0,48 |
| rpsO (S15) | 89 | 2,15 | 43200 | 5,82 | 2,71 | 5 | 0,54 |
| rpsP (S16) | 82 | 2,04 | 43200 | 5,42 | 2,66 | 4,3 | 0,62 |
| rpsQ (S17) | 84 | 2,06 | 43200 | 5,49 | 2,66 | 4,9 | 0,54 |
| rpsR (S18) | 75 | 1,89 | 43200 | 4,89 | 2,59 | 4,7 | 0,55 |
| rpsS (S19) | 92 | 2,2 | 43200 | 6,02 | 2,73 | 5,3 | 0,52 |
| rpsT (S20) | 87 | 2,11 | 43200 | 5,68 | 2,69 | 6,5 | 0,41 |
| rpsU (S21) | 71 | 1,82 | 43200 | 4,63 | 2,49 | 3,9 | 0,64 |

*μ*=0.42 h^-1^ *n*_R(av)_ = 6800 *ε*_aa(av)_ = 43200 amino acids h^-1^ [15]
