# Supplementary material for: Measurement of the Rates of Synthesis of Three Components of Ribosomes of Mycobacterium fortuitum: A Theoretical Approach to qRT-PCR Experimentation
Source: PLoS One. 2010 Jul 14;5(7):e11575. doi: 10.1371/journal.pone.0011575 (PMC2904383; doi:10.1371/journal.pone.0011575)
Supplement: Table S2 — Synthesis of proteins of the 50S subunit of E.coli ribosomes calculated on the basis of transcription/translation coupling (see equations presented in Table 2). The abbreviations are defined in Table 1. lt ntr(i)(av) defines the limit (lt) to the numbers of transcripts per population average cell. The last column on the right is the fraction of the limiting (lt) number of transcripts needed to synthesize the required number (6800) of copies of the protein specified. The limiting number of transcripts was estimated from the product of the number of copies of the specified ORF per cell and the maximum number (3 laa(i)/80) of transcripts per ORF. The number of copies of a particular ORF per cell was obtained by obtained by means of equation (9) of reference 15. Data for rplJ and rplL are explained in the main text. (0.09 MB DOC) [file pone.0011575.s002.doc]

**Table S2. Synthesis of proteins of the 50S subunit of *E.coli* ribosomes calculated on the basis of transcription/translation coupling (see equations presented in Table 2).**

| r-protein | *l*_aa(i)_ | *n*_R(i)/tr(i)_ | *ε*_aa(av)_ | *n*_R(i)(av)_ | *n*_tr(i)(av)_ | *lt n*_tr(i)(av)_ | *n*_tr(i)(av)/_ *lt n*_tr(i)(av)_ |
| --- | --- | --- | --- | --- | --- | --- | --- |
| rplA (L1) | 234 | 4,89 | 43200 | 15,47 | 3,16 | 15,7 | 0,2 |
| rplB (L2) | 273 | 5,62 | 43200 | 18,05 | 3,21 | 15,76 | 0,2 |
| rplC (L3) | 209 | 4,42 | 43200 | 13,81 | 3,13 | 12,1 | 0,26 |
| rplD (L4) | 201 | 4,27 | 43200 | 13,29 | 3,11 | 11,6 | 0,27 |
| rplE (L5) | 179 | 3,86 | 43200 | 11,83 | 3,07 | 10,34 | 0,3 |
| rplF (L6) | 177 | 3,82 | 43200 | 11,7 | 3,06 | 10,22 | 0,3 |
| rplI (L9) | 149 | 3,29 | 43200 | 9,85 | 2,99 | 9,39 | 0,32 |
| rplJ (L10) | 165 | 3,59 | 86400 | 21,82 | 6,08 | 11,14 | 0,54 |
| rplK (L11) | 142 | 3,16 | 43200 | 9,38 | 2,97 | 9,6 | 0,31 |
| rplL (L12) | 121 | 2,77 | 86400 | 16 | 5,8 | 8,19 | 0,71 |
| rplM (L13) | 429 | 8,54 | 43200 | 28,36 | 3,32 | 24,83 | 0,13 |
| rplN (L14) | 123 | 2,81 | 43200 | 8,13 | 2,89 | 7,16 | 0,4 |
| rplO (L15) | 144 | 3,2 | 43200 | 9,52 | 2,97 | 8,37 | 0,35 |
| rplP (L16) | 136 | 3,05 | 43200 | 8,99 | 2,95 | 7,91 | 0,37 |
| rplQ (L17) | 127 | 2,88 | 43200 | 8,39 | 2,91 | 7,39 | 0,39 |
| rplR (L18) | 117 | 2,69 | 43200 | 7,73 | 2,87 | 6,81 | 0,42 |
| rplS (L19) | 115 | 2,66 | 43200 | 7,6 | 2,86 | 6 | 0,48 |
| rplT (L20) | 118 | 2,71 | 43200 | 7,8 | 2,88 | 6,11 | 0,47 |
| rplU (L21) | 103 | 2,43 | 43200 | 6,81 | 2,8 | 5,85 | 0,48 |
| rplV (L22) | 110 | 2,56 | 43200 | 7,27 | 2,84 | 6,41 | 0,44 |
| rplW (L23) | 100 | 2,38 | 43200 | 6,61 | 2,78 | 5,83 | 0,48 |
| rplX (L24) | 104 | 2,45 | 43200 | 6,87 | 2,8 | 6,06 | 0,46 |
| rplY (L25) | 94 | 2,26 | 43200 | 6,21 | 2,75 | 4,6 | 0,6 |
| rpmA(L27) | 85 | 2,09 | 43200 | 5,62 | 2,69 | 4,97 | 0,54 |
| rpmB(L28**)** | 78 | 1,96 | 43200 | 5,16 | 2,63 | 4,92 | 0,53 |
| rpmC(L29) | 63 | 1,68 | 43200 | 4,16 | 2,48 | 3,7 | 0,67 |
| rpmD(L30) | 59 | 1,61 | 43200 | 3,9 | 2,42 | 3,47 | 0,7 |
| rpmE(L31) | 70 | 1,81 | 43200 | 4,63 | 2,56 | 4,69 | 0,55 |
| rpmF(L32) | 57 | 1,57 | 43200 | 3,77 | 2,4 | 3,37 | 0,71 |
| rpmG(L33) | 55 | 1,53 | 43200 | 3,64 | 2,34 | 3,49 | 0,67 |
| rpmH(L34) | 46 | 1,36 | 43200 | 3,04 | 2,23 | 2,96 | 0,75 |
| rpmI(L35) | 65 | 1,72 | 43200 | 4,3 | 2,5 | 3,39 | 0,74 |
| rpmJ(L36) | 117 | 2,69 | 43200 | 7,73 | 2,87 | 6,81 | 0,42 |

*μ*=0.42 h^-1^ *n*_R(av)_ = 6800 *ε*_aa(av)_ = 43200 amino acids h^-1^ [15]
