# Supplementary material for: Measurement of the Rates of Synthesis of Three Components of Ribosomes of Mycobacterium fortuitum: A Theoretical Approach to qRT-PCR Experimentation
Source: PLoS One. 2010 Jul 14;5(7):e11575. doi: 10.1371/journal.pone.0011575 (PMC2904383; doi:10.1371/journal.pone.0011575)
Supplement: Table S3 — The Shine/Dalgarno motifs of rplJ (A) and the intergene regions (B) separating rplJ and rplL of representative species of Mycobacterium. Sequences upstream from rplJ and intergene sequences are shown by lower case letters. The Shine/Dalgarno motifs and their binding sites are shown in bold italics. The light shading indicates secondary Shine/Dalgarno motifs. (0.06 MB DOC) [file pone.0011575.s003.doc]

**Table S3. The Shine/Dalgarno motifs of *rpl*J (A) and the intergene regions (B) *s*eparating *rpl*J *and rpl*L of representative species of *Mycobacterium*.**

A

| Species |  | *rpl*J |
| --- | --- | --- |
| *M.leprae* TN | 5’cctc***aggagg*** | tatgcATG3’ |
| *M.tuberculosis* H37Rv | 5’cgtc***aggagg*** | tatgcATG3’ |
| *M.gilvum* PYR-GCK | 5’cacg***aggagg*** | caagcATG3’ |
| *M.avium* 104 | 5’gagc***aggagg*** | taggcATG3’ |
| Anti-Shine/Dalgarno motif | 3’ucuu***uccucc***5**’** |  |

B

| Species | *rpl*J |  |  |  |  | *rpl*L |
| --- | --- | --- | --- | --- | --- | --- |
| *M.leprae* TN | 5’TAG | ccaagcacca | *---------*a | gaaaatca***ag*** | ***gaagga***cccc | acccccATG3’ |
| *M.tuberculosis* H37Rv (a) | 5’TAG | tcacccagca | ccccaca--- | ------cc***ag*** | ***gaagga***ccgc | ccatc-ATG3’ |
| *M.gilvum* PYR-GCK (b) | 5’TAG | taccaagacc | accca-agaa | gtaa--ta**ag** | ***gaagga***ccat | caac--ATG3’ |
| *M.avium* 104 (c) | 5’TAA | ccaccaaacc | cggaacttgg | gaagaaca**ag** | ***gaagga***ccca | caccc-ATG3’ |
| Anti-Shine/Dalgarno motif (d) |  |  |  | **3’*uc*** | ***uuuccu****cc***5’** |  |

(a),representative of all members of the *M.tuberculosis* complex;

(b),representative of *M.vanbaalenii* PYR-1*, Mycobacterium sp.*JLS *,Mycobacterium sp.*KMS*, Mycobacterium sp.*MCS*;*

(c),representative of *M.avium subsp.*paratuberculosis K-10;

(d),Kempsell *et al.,*1992 (reference 27)*.*
